# Outcomes of Two Different Obstetric Social Reintegration Program Approaches After Obstetric-Related Fistula Repair

**DOI:** 10.1007/s00192-025-06361-1

**Published:** 2025-11-12

**Authors:** Itengré Ouedraogo, Priti Dutta, Holly E. Richter, Lynda Ugwu, Loada Landry

**Affiliations:** 1 Association Renaissance Fistula Center, Ougadougou, Burkina Faso; 2 Division Urogynecology and Reconstructive Pelvic Surgery, University of Alabama at Birmingham, 1700 6th Av South, Suite 10382, Birmingham, AL USA

**Keywords:** Fistula center, Psychosocial and economic outcomes, Reintegration programs, Vesicovaginal fistula repair

## Abstract

**Introduction and Hypothesis:**

The objective was to characterize psychosocial–economic outcomes after obstetric-related vesicovaginal fistula repair (OFR) and completing a comprehensive psychosocial–economic reintegration service at a dedicated fistula center (Association Renaissance, ARENA) compared with women receiving both services in a multispecialty country hospital (Larry Ebert Medical Center, LEMC).

**Materials and Methods:**

This retrospective study included women who underwent OFR and re-integration training at ARENA fistula center, Ouagadougou, Burkina Faso, from January 2022 through July 2023, and at LEMC from July 2020 through March 2022. A standardized questionnaire assessing areas of household/income/employment/finance experiences, support resources, and patients’ feelings was completed by in-person interview or phone. Analyses included Wilcoxon’s rank sum tests, Chi-squared, or Fisher’s exact test. Logistic regression models adjusting for age, time living with fistula, and time from surgery were performed to evaluate associations of selected outcomes.

**Results:**

Median age and time having a fistula between the ARENA center (*N*=134) and LEMC (*N*=146) participants were similar: (age, years, median, [IQR] 32.0 [25, 40] vs 34.0 [27, 41] respectively, *p* = 0.30; time with fistula, years 1.6 [0.2, 10.0] vs 2.5 [0.8, 11] respectively, *p* = 0.06). Women undergoing treatment at a dedicated fistula center had greater odds of self-satisfaction (aOR4.6, 95% CI 1.4,15.2), being a person of worth (aOR 12.6, 95% CI 3.7, 42.4), and having income-generating assets (aOR 5.1, 95% CI 1.7, 14.9).

**Conclusions:**

Overall, psychosocial and economic outcomes for women after OFR reflected greater improvement when post-OFR reintegration services were at a dedicated fistula center.

**Supplementary Information:**

The online version contains supplementary material available at 10.1007/s00192-025-06361-1

## Introduction

For a young woman suffering obstructive obstetric complications, the reality may be days of labor before she can reach the nearest hospital, potentially developing pelvic floor morbidity including vesicovaginal fistula (VVF) and/or rectovaginal fistula (RVF). Should she survive the trauma from that birth with a fistula, she may have a dead baby and face ostracization from her family and society, as the shame from incontinence affects her ability to work and perform household/marital functions. Women in rural Burkina Faso have limited knowledge in preventing these fistulas, so there is a great need and recognition that programs not only address the repair of the obstetric-related vesicovaginal and/or rectovaginal fistula (OFR) itself but also facilitate reintegration into society in a productive manner, with potentially improved psychosocial and economic interactions [1]. Other medical sequelae including musculoskeletal, pelvic floor morbidity, reproductive issues, and sexual function also contribute to post-OFR quality of life and should be addressed [2]. Because of the impact that an obstetric fistula can have on a woman’s sense of self and her place in society, simply repairing the fistula is often not enough. Although fistula repair can improve a woman’s physical capabilities, these women continue to face challenges reconnecting with their community, economic hardship, and marital issues [3–7].

It has been recognized that effective reintegration programs addressing individualized needs increase survivors’ self-esteem and mental health, reduce stigma, and facilitate social acceptance, including economic independence and other family expectations and employment barriers [8, 9]. With support from various international organizations, many African countries have developed National Obstetric Fistula Strategic Frameworks to create centers that not only repair the fistula but also repair patients’ sense of self-worth [10–13]. One such center is the Association Renaissance (ARENA) Fistula Center in Ouagadougou, Burkina Faso, which offers dedicated ORF repair and holistic psychosocial–economic reintegration services. The primary aim of this current study was to evaluate psychosocial and economic outcomes of women who undergo the services offered by a dedicated fistula center (ARENA) compared with a multispecialty country hospital (Larry Ebert Medical Center, LEMC). Our hypothesis was that undergoing reintegration training in a dedicated fistula center would provide more robust outcomes than that in a multispecialty surgery center. As a secondary aim, changes in psychosocial–economic consequences within the dedicated fistula center (ARENA) reintegration program were investigated.

## Materials and Methods

### Study Settings

The ARENA fistula center is a medical center dedicated to the care of women affected by obstetric fistula in Ouagadougou, Burkina Faso. It has a capacity of 25 beds, accommodation for women, and a unit dedicated to more formalized social reintegration services. The women receive social reintegration services, including education, psychological care, financial support for existing activities, and more diversified training in income-generating activities such as: liquid and solid soap manufacturing, karite butter and sesame cookie manufacturing, the basics of animal breeding, beadwork, embroidery, and weaving. Women were also empowered through training in basic literacy, basic accounting for small businesses, and education in sexual and reproductive health. Besides equipping them for their own sexual and reproductive lives, this makes them advocacy agents for women’s health in their communities. The reintegration program is usually initiated post-surgery as they wait for catheter removal and lasts 4–6 weeks depending on the patient’s desire.

The LEMC is a multidisciplinary medical center offering maternal and child health care, general medicine, and specialties including surgery. ARENA providers were granted a 10-bed ward dedicated to women with obstetric fistula who, after surgery, had the possibility of receiving social reintegration services during their convalescence. These services were mainly education, assistance with basic needs, and short training in income-generating activities (making liquid soap) lasting for 4–6 weeks, which could be influenced by bed availability.

### Study Population

The study population included women with vesicovaginal fistulas treated at the LEMC from July 2020 to December 2021 and completing social reintegration services, and those treated at Centre Medical Renaissance (ARENA fistula center) between June 2022 and July 2023 and completing social reintegration services.

### Methodology

Institutional Review Board Approval was obtained at the US site. As the risks in the proposed study were anticipated to be minimal and within the standard of current clinical care, the ARENA Clinical Oversight & Ethics Committee comprising staff, stakeholders, and ARENA Board Members approved the project.

### Data Collection

A standardized questionnaire administered pre- and post-OFR surgery addressing areas of household, income/employment/finances, support resources, and “patients feelings” was designed (Appendix 1). It was designed by the first and senior authors after the women at the Larry Ebert facility received their surgery. Initially created in French, it was translated into English for analytical purposes (see Appendix). A team of interviewers comprising community mobilizers and nurses was set up and trained in the use of the questionnaire. To limit language difficulties, local language (Moore, Fulfulde, Dioula) translators were used. The interviewers presented the content and purpose of the interview to the participant, and offered her informed consent, which she had the right to accept or refuse.

Patients treated at the multidisciplinary setting, patient characteristics, and post-service assessment data were compiled by interviewers reaching out to patients in their communities or during clinical follow-up and by telephone when it was not possible to reach out. Data collection was completed by review of medical records.

Patients treated at the ARENA dedicated center completed a standardized pre-service assessment questionnaire by interviewers. The post-services assessments (surgery and social reintegration services) data were collected by a combination of clinical follow-up visits, in the field in women’s home villages, or by cell phone. This allowed direct pre- and post-operative comparison for this group. A review of medical records completed data collection.

### Data Analysis

Data were analyzed using Wilcoxon’s Rank sum tests for categorical variables, and Chi-squared or Fisher’s exact tests for continuous variables. Frequencies (*n* and percentages) and medians and interquartile ranges were reported. After a review of patient responses, five outcomes were chosen to explore associations between site and self-awareness, socialization, community relationships, and income-generating assets (i.e., I feel that I am a person of worth, Ability to socialize, Relationship with community, and Ownership of income-generating assets). Logistic regression models were used to adjust for age, time living with a fistula, and time from surgery to interview to evaluate the associations and report adjusted odds ratios and 95% confidence limits. McNemar’s test was used to assess the pre- and post-surgery responses of ARENA participants. Analyses were performed using SAS software 9.4.

## Results

### Comparative Reintegration Outcomes Between Centers

Of 165 patients treated in the multidisciplinary setting (LEMC), 146 (88.5%) completed the post-surgery assessment measure. Of 200 patients who completed the pre-service assessment tool at the dedicated center (ARENA), 134 (67%) completed the post-service assessment tool and were included in the study. Reasons for the other *n* = 66 subjects not completing the post-service/surgery assessment tool included, 59 out of 66 (90%) were restricted by country security issues preventing the patients from returning to the center and prevented the mobilization team from reaching the villages. A smaller proportion 7 out of 66 (10%) could not be reached by cell phone. Clinico-demographics of participants are noted in Table 1. Median age at presentation and time in years spent living with the fistula prior to repair were similar between groups (age in years, median [IQR] ARENA 32.5 [25, 40] vs LEMC 34.0 [27, 41], *p* = 0.30; time with fistula in years ARENA 1.6 [0.2, 10.0] vs LEMC 2.5 [0.8, 11], *p* = 0.06). Median time from surgery to interview was significantly shorter for participants at ARENA than those at LEMC (median [IQR], ARENA 6.1 months [6.0, 6.7] vs LEMC 18.0 months [13.7, 21.5], *p*<0.001). Post-surgical repair, the ARENA patients were more likely to own income-generating assets than those from LEMC (35.1% ownership vs 15.7%, *p* < 0.001; Table 2). ARENA patients were also more likely to own businesses post-repair (41% vs 9.6%, *p* < 0.001) whereas LEMC patients were more likely to be housewives post-repair (18.7% vs 69.9%, *p* < 0.001). A similar proportion of participants received family-planning services and emotional support from both institutions ([ARENA, LEMC]; family planning [86.6%, 88.4%, *p*=0.65]; emotional support [99.2, 100.0, *p* = 0.47]; Table 3). However, significantly more women received education, business training/opportunities, and financial assistance from ARENA than from LEMC (education: 99.2% vs 28.1%, *p* < 0.001; business training: 74.6% vs 11%, *p* < 0.001; financial assistance: 58.3% vs 24.7%, *p* < 0.001). In contrast, more women received material needs such as food and clothing from LEMC than from ARENA (95.2% vs 78.4%, *p* < 0.001).

**Table 1:** Characteristics of reintegration program participants

	ARENA (*N* = 134)	Larry Ebert (*N* = 146)	*p* value
Age, years			0.30
Mean (SD)	34.3 (13.7)	34.7 (11.5)	
Median IQR	32.5 (25, 40)	34 (27, 41)	
Time living with fistula, years			
Mean (SD)	7.02 (10.0)	6.6 (8.5)	0.06
Median IQR	1.6 (0.2, 10.0)	2.5 (0.8, 11)	
Time from surgery to interview			
Mean (SD)	6.5 (1.6)	16.9 (5.8)	0.0001
Median (IQR)	6.1. (6.0, 6.7)	18.0 (13.7, 21.5)	

*SD* standard deviation, *IQR* interquartile range, *ARENA* Association Renaissance

*p* values based on Kruskal–Wallis test

**Table 2:** Post-surgery comparative assessment of household, employment, and finances

	ARENA (*N* = 134)	Larry Ebert (*N* = 146)	*p* value
Marital status			0.007
Married	64 (47.8)	96 (65.7)	
Separated/divorced/widowed	61 (45.5)	46 (31.5)	
Single	9 (6.7)	4 (2.7)	
Whom do you live with			0.006
Husband	67 (50.0)	91 (62.3)	
Parents	34 (25.4)	40 (27.4)	
Other	33 (24.6)	15 (10.3)	
Profession			<0.001
Business	55 (41.0)	14 (9.6)	
Farmer	25 (18.7)	9 (6.2)	
Housewife	25 (18.7)	102 (69.9)	
Laborer	8 (6.0)	1 (0.7)	
Other	21 (15.7)	20 (13.7)	
Primary financial support			<0.001
Self	47 (35.1)	41 (28.1)	
Husband	41 (30.6)	80 (54.8)	
Parents	25 (18.7)	22 (15.1)	
Other	21 (15.7)	3 (2.1)	
Do you own income-generating assets	47 (35.1)	23 (15.7)	<0.001
Used savings for business/money lending	11 (8.2)	6 (4.1)	0.15
Expenses covered by own income			0.003
At least half to all	62 (46.3)	62 (42.3)	
Less than half	45 (33.6)	72 (49.3)	
None	27 (20.1)	12 (8.2)	

*ARENA* Association Renaissance

*p* values based on Chi-squared test or Fisher’s exact test

**Table 3:** Post-surgery comparative assessment of support resources, family planning and physical limitations

	ARENA (*N* = 134)	Larry Ebert (*N* = 146)	*p* value
Type of support			
Education or learning a skill	133 (99.2)	41 (28.1)	<.001
Business training or opportunities	100 (74.6)	16 (11.0)	<.001
Financial assistance	77/132 (58.3)	36 (24.7)	<.001
Material needs such as food or clothing	105 (78.4)	139 (95.2)	<.001
Shelter	97 (72.4)	–	–
Family planning counseling or services	116 (86.6)	129 (88.4)	0.65
Emotional or psychological support	133 (99.2)	146 (100.0)	0.47
Joining a support or solidarity group	45 (33.6)	18 (12.3)	<.001
Support most benefited from			
Education or learning a skill	132 (98.5)	36 (24.7)	<.001
Business training or opportunities	76 (56.7)	15 (10.3)	<.001
Financial assistance	52 (38.8)	35 (24.0)	0.007
Material needs food or clothing	56 (41.8)	131 (89.8)	<.001
Shelter	25 (18.9)	–	–
Family planning counseling or services	78 (58.2)	106 (72.6)	0.01
Emotional or psychological support	–	–	–
Joining a support or solidarity group	37 (27.6)	18 (12.3)	0.001
Physical limitations			
Difficulty walking?	4 (3.0)	5 (3.4)	0.83
Family planning			
Any birth control/family planning	36 (26.9)	27 (18.5)	0.09

*ARENA* Association Renaissance

*p* values based on Chi-squared test or Fisher’s exact test

Reviewing services offered in both institutions, significantly more women felt that they received the most benefit from education and business training in ARENA than participants at LEMC (education: 98.5% vs 24.7%, *p* <.001; business training: 56.7% vs 10.3%, *p* < 0.001; Table 3). However, more LEMC patients felt that they benefited most from material needs than at ARENA (89.8% vs 41.8%, *p* < 0.001).

Patients at ARENA were more likely to be satisfied with themselves (95.5% vs 78.1%, *p* < 0.001) and feel of equal worth to others (96.3% vs 84.2%, *p* < 0.001) than patients at LEMC (Table 4). Additionally, they were less likely to feel that they were “no good at all” (2.2% vs 12.3%, *p* = 0.001) or “do not have much to be proud of” (3.0% vs 20.0%, *p* < 0.001) than LEMC participants. Good or excellent relationships with the community (91.8% vs 76.0%, *p* < 0.001), ability to socialize (91.8% vs 76.0%, *p* < 0.001), family life (83.6% vs 67.1%, *p* < 0.001), and being very optimistic about the future (82.1% vs 56.2%, *p* < 0.001) were greater in the ARENA than in the LEMC participants. Other specific items assessed are noted in Tables 2, 3, and 4. Participants undergoing treatment at the dedicated fistula center had greater odds of self-satisfaction, being a person of worth, and having income-generating assets (Table 5).

**Table 4:** Post-surgery comparative assessment of patient’s feelings, ability to work, and community relationship

		ARENA (*N* = 134)	Larry Ebert (*N* = 146)	*p* value
Self-evaluation				
I am satisfied with myself	128 (95.5)		114 (78.1)	<0.001
At times I feel like I am no good at all	3 (2.2)		18 (12.3)	0.001
I can do things as well as most other people	121 (90.3)		126 (86.3)	0.30
I feel I do not have much to be proud of	4 (3.0)		29 (20.0)	<0.001
I am inclined to feel that I am a failure	3 (2.2)		5 (3.4)	0.72
I feel that I am a person of worth at least equal with others	129 (96.3)		123 (84.2)	<0.001
Marriage				0.01
Poor/fair	47 (35.1)		51 (34.9)	
Good/excellent	53 (39.5)		77 (52.7)	
N/A	34 (25.4)		18 (12.3)	
Family life				<0.001
Poor/fair	17 (12.7)		48 (32.9)	
Good/excellent	112 (83.6)		98 (67.1)	
N/A	5 (3.7)		0	
Ability to do housework				0.003
Poor/fair	25 (18.7)		10 (6.9)	
Good/excellent	109 (81.3)		136 (93.1)	
Ability to do activities outside the home				0.11
Poor/fair	27 (20.1)		19 (13.0)	
Good/excellent	107 (79.8)		127 (87.0)	
Ability to attend school or work				0.002
Poor/fair	30 (22.4)		44 (30.1)	
Good/excellent	95 (70.9)		102 (69.9)	
N/A	9 (6.7)		0	
Ability to socialize				<0.001
Poor/fair	11 (8.2)		35 (24.0)	
Good/excellent	123 (91.8)		111 (76.0)	
Relationship with community				<0.001
Poor/fair	11 (8.2)		33 (22.6)	
Good/excellent	123 (91.8)		113 (77.4)	
Little interest or pleasure in doing things in the past 2 weeks				0.002
More than half to all days	0		10 (6.8)	
Several days to none	134 (100.0)		136 (93.1)	
Down, depressed or hopeless in the past 2 weeks				0.002
More than half to all days	0		10 (6.8)	
Several days to none	133 (100.0)		136 (93.1)	
Hopefulness about future				<0.001
Somewhat	24 (17.9)		64 (43.8)	
Very optimistic	110 (82.1)		82 (56.2)	

*ARENA* Association Renaissance

*p* values based on Chi-squared or Fisher’s exact test

**Table 5:** Post-surgery comparative logistic regression for select characteristics

	ARENA, *n* (%)	Larry Ebert, *n* (%)	Adjusted odds ratio (95% confidence interval)
I am satisfied with myself	128 (95.5)	114 (78.1)	4.6 (1.4, 15.2)
I am a person of worth	129 (96.3)	123 (84.2)	12.6 (3.7, 42.4)
Ability to socialize (good/excellent)	123 (91.8)	111 (76.0)	1.7 (0.6, 5.2)
Relationship with community (good/excellent)	123 (91.8)	113 (77.4)	1.6 (0.5, 5.2)
Income-generating assets	47 (35.1)	23 (15.7)	5.1 (1.7, 14.9)

Models adjusted for age, time living with fistula, and time from surgery to interview

*ARENA* Association Renaissance

## Pre- and Post-Surgical Outcomes: Association Renaissance Dedicated Fistula Center

As was the nature of this retrospective research study, baseline questionnaires were not performed in the comparative analyses groups; therefore, it was thought that reporting of baseline assessments and change after fistula repair at the ARENA dedicated fistula center would provide important perspective regarding magnitude of changes seen. A total of 134 participants completed pre- and post-surgery interviews. Median age was 32.5 years (IQR 25, 40), median years living with the fistula prior to repair was 1.6 (IQR 0.2, 10.0), and median time from surgery to interview was 6.1 months (IQR 6.0, 6.7). Patients were more likely to resume living with their husbands and less likely to live with parents after surgery (living with husband pre-surgery 38.1% vs post-surgery 50.0%, *p* < 0.001; living with parent 41.0% pre-surgery vs 25.4% post-surgery, *p* < 0.001; Table 6). Prior to surgery, 9% of patients worked in business, which increased to 41% after surgery. Additionally, fewer women were housewives and farmers after their fistula repair (housewives 30.6% to 18.6%, farmers 35.8% to 18.6%).

**Table 6:** Association Renaissance (ARENA) dedicated fistula center pre-/post-obstetrics-related fistula repair outcomes (*N*=134)

	Pre-surgery, *n* (%)	Post-surgery, *n* (%)	*p* value
Household and income			
Marital status			0.01
Married	50 (37.3)	64 (47.8)	
Separated/divorced/widowed	75 (56.0)	61 (45.5)	
Single	9 (6.7)	9 (6.7)	
Whom do you live with?			<0.001
Husband	51 (38.1)	67 (50.0)	
Parents	55 (41.0)	34 (25.4)	
Other	28 (20.9)	33 (24.6)	
Profession			<0.001
Business	12 (9.0)	55 (41.0)	
Farmer	48 (35.8)	25 (18.6)	
Housewife	41 (30.6)	25 (18.6)	
Laborer	2 (1.5)	8 (6.0)	
Student	32 (23.9)	21 (15.6)	
Main financial support			<0.001
Self	25 (18.7)	47 (35.1)	
Husband/partner	38 (28.4)	41 (30.6)	
Parents	53 (39.5)	25 (18.7)	
Other	18 (13.4)	21 (15.7)	
Do you own income-generating assets	16 (11.9)	47 (35.1)	<0.001
Used savings for business/money lending	2 (1.5)	11 (8.2)	0.007
Expenses covered by own income			<0.001
At least half to all	16 (11.9)	62 (46.3)	
Less than half	66 (49.2)	45 (33.6)	
None	52 (38.8)	27 (20.1)	
Difficulty walking, family planning (*n* = 134)			
Difficulty walking?	25 (18.7)	4 (3.0)	<0.001
Any birth control/family planning	10 (7.5)	36 (26.9)	<0.001
ARENA pre-/post-surgery, patient’s feelings (*n* = 134)			
Self-evaluation			
At times I feel like I am no good at all	121 (90.3)	3 (2.2)	<0.001
I can do things as well as most other people	17 (12.7)	121 (90.3)	<0.001
I feel I do not have much to be proud of	96 (71.6)	4 (3.0)	<0.001
I am inclined to feel that I am a failure	96 (71.6)	3 (2.2)	<0.001
I feel that I am a person of worth at least equal with others	51 (38.1)	129 (96.3)	<0.001
Marriage			
Poor/fair	104 (77.6)	47 (35.1)	<0.001
Good/excellent	12 (9.0)	53 (40.0)	
N/A	18 (13.4)	34 (25.4)	
Family life			–
Poor/fair	109 (81.3)	17 (12.7)	
Good/excellent	25 (18.7)	112 (83.6)	
N/A	0	5 (3.7)	
Ability to do housework			–
Poor/fair	128 (95.5)	25 (18.7)	
Good/excellent	5 (3.7)	109 (81.3)	
N/A	1 (1.0)	0	
Ability to do activities outside the home			<0.001
Poor/fair	133 (99.3)	27 (20.1)	
Good/excellent	1 (1.0)	107 (80.0)	
Ability to attend school or work			<0.001
Poor/fair	107 (79.9)	30 (22.4)	
Good/excellent	1 (1.0)	95 (70.9)	
N/A	26 (19.4)	9 (6.7)	
Ability to socialize			<0.001
Poor/fair	128 (95.5)	11 (8.2)	
Good/excellent	6 (4.5)	123 (91.8)	
Relationship with community			–
Poor/fair	134 (100.0)	11 (8.2)	
Good/excellent	0	123 (91.8)	
Little interest or pleasure in doing things in the past 2 weeks			
More than half to all days	82 (61.2)	134 (100.0)	
Several days to none	52 (38.8)	0	
Down, depressed or hopeless in the past 2 weeks			
More than half to all days	111 (83.5)	133 (100.0)	
Several days to none	22 (16.5)	0	
Hopefulness about future			
Not at all	134 (100.0)	24 (17.9)	
Very optimistic	0	110 (82.1)	

*N/A* not available

*p* values based on the McNemar–Bowker test

Participants were more likely to support themselves financially and less likely to rely on parents post-repair. Pre-surgery, 90.3% of patients felt that they were “no good at all” and 71.6% felt they were “a failure,” which decreased dramatically to 2.2% (*p* < 0.001) and 2.2% (*p* < 0.001) respectively after reintegration services. Their perception that they can “do things as well as most other people” and are “of worth at least equal with others” increased dramatically (12.7% to 90.3%, *p* < 0.001) and 38.1% to 96.3%, *p* < 0.001 respectively). There was a statistically significant perceived improvement of their marriages, ability to attend school/work, and ability to socialize after surgery (*p* < 0.001; Table 6). Pre-surgery, all patients reported a poor/fair relationship with the community, which improved to 91.8% of patients having a good/excellent relationship after reintegration services. All 134 patients reported not feeling hopeful about the future prior to surgery. However, after surgery and the reintegration services, 82.1% of them felt “very optimistic” about the future (Table 6).

## Discussion

This retrospective cohort study (with a prospective component) showed that in several areas of psychosocial–economic assessments, a greater proportion of women cared for at a dedicated fistula center reported outcomes better than those undergoing surgery at a multispecialty medical center. Specifically, ARENA patients were significantly more likely to have a positive self-evaluation of themselves and improved family life, marriage, mental health, and relationship with the community than those treated at the multispecialty hospital. ARENA patients also had greater access to education, business training, and financial assistance. In some interesting areas there were no differences, specifically in family-planning counseling and emotional and psychological support. In other areas, there appeared to be greater benefit at the multispecialty center, such as providing food/clothing for patients. However, importantly, these data do not reflect a change in baseline assessments as they had not been performed in the country hospital.

A study of Ethiopian women post-fistula repair identified post-repair interventions that would help optimize quality of life. These included post-repair counseling about fistula and risk factors associated with recurrence, community-based follow-up, linkages to income-generating opportunities, engagement of affected women for community outreach and metrics for evaluating rehabilitation and social reintegration efforts to help attain productive lives [14]. Since this study, as noted above, many countries have recognized the importance of supportive treatments and the United Nations Population Fund (UNFPA) recognizes this as a “pillar” of the global roadmap to end obstetric fistula [15]. These current data add to the body of knowledge with respect to structuring a holistic reintegration program.

The current investigation primarily focused on assessing the outcomes of patients at a dedicated fistula center (ARENA) compared with those women receiving care in a multispecialty hospital (LEMC). Compared with patients from fistula-specific repair centers in Ethiopia and Kenya, whose patients reported improved self-esteem, our patients also had significantly improved mental health and outlook. These studies found that the peer support from other patients with a fistula played a significant role in improvement in these areas [3, 16]. Additionally, the current patients experienced a significantly improved relationship with the community, but 8.2% of ARENA patients continued to have a fair/poor relationship with the community. Fistula repair centers in Ethiopia, Kenya, and Nigeria also had the vast majority of patients experience a significantly improved relationship with the community. But they also had some patients who did not, owing to lingering pain, unsuccessful repairs, and continued stigmatization, which may also play a role in our finding [3, 16, 17].

A study including patients who had fistulas repaired at multispecialty hospitals in Uganda found that after surgery, many women often experienced worse economic situations owing to having to use savings, sell income-generating assets, and other experiences of economic exploitation [18]. However, many women from fistula-repair programs throughout the continent experience improved economic outcomes after surgery owing to the resources that they receive in order to own property and generate income [3, 14]. This is a difference that the current data reflect as well. Income-generating assets for patients who had their fistulas repaired at ARENA increased from 11.9% pre-operatively to 35.1% post-operatively, whereas the income-generating assets of LEMC patients decreased from 35.1% to 15.7% respectively.

With regard to the ARENA pre/post data there are evident improvements in patients’ self-evaluation of themselves, perception of their marriages, and their family lives. Additionally, there is substantial progress in ARENA patients’ abilities to do housework, activities outside of the home, attend school/work, and socialize. Prior to surgery, all ARENA patients reported a poor/fair relationship with the community. However, after surgery, 91.8% of those patients ended up with a good/excellent relationship with the community.

There are fistula repair programs similar to ARENA throughout the African continent, with different approaches to tackling fistula repair and social reintegration. Based on a systematic review of social rehabilitation practices throughout Africa, Uganda, Nigeria, Tanzania, and Guinea have been recognized for the implementation of best practices in fistula-repair reintegration services [10]. However, as also noted in this systematic review, multiple countries in sub-Saharan Africa still struggle with the implementation of rehabilitation programs owing to a lack of personnel and infrastructure [10]. Programs in Nigeria are similar to ARENA, in which they offer skills training, health education, and material items for home-based care [10]. However, programs in Uganda and Guinea have a greater impact on the community, focusing on eliminating ostracism/stigma, training patients to be “Fistula Ambassadors” to promote awareness, and encouraging volunteers in the community to house and provide support for fistula patients who have been rejected by their community [10]. ARENA focuses more on providing support and education for individual patient empowerment, rather than community initiatives to decrease stigmatization. For example, the majority of ARENA patients receive education, business training, financial assistance, material needs, and family-planning services. This is reflected in the fact that ARENA patients who worked in business increased from 9% pre-operatively to 41% post-operatively and were less likely to be housewives/farmers after surgery.

Limitations of this study include that baseline assessments were unavailable in the multispecialty country hospital treatment center as the questionnaire had not been developed at that time; thus, differences in the magnitude of changes could not be assessed. Time to follow-up differed between the centers, where more time had elapsed since surgery in those from the multispecialty hospital, potentially providing a more stable outcome. Additionally, standardized questionnaires were utilized but not validated by an external body or qualitative analysis. Differences in symptom severity and fistula type were not captured on medical record review and may have impacted baseline and post-operative responses. Questionnaires filled out by patients consisted only of subjective evaluations of their situations and were not complemented by objective data. Last, analyses did not control for multiple comparisons.

Strengths include that the existence of community-based outcome assessors made this study feasible. Robust follow-up of participants who underwent fistula repair during the noted time periods mitigated potential bias from nonresponsive patients. Finally, the questionnaires assessed a significant amount of detail in the areas of economic, psychological, social, mental, and physical health assessments.

In summary, overall post-obstetric fistula repair outcomes from a holistic reintegration program at a dedicated fistula center may be more robust than those achieved in a multidisciplinary medical center setting. As increased efforts to treat obstetric-related fistula continue, the surgical infrastructure setting should be considered.

## Supplementary Information

Below is the link to the electronic supplementary material.

Supplementary file1 (DOCX 58 KB)

## Data Availability

Data may be obtained by contacting the corresponding author.
