# Supplementary material for: Outcomes of Two Different Obstetric Social Reintegration Program Approaches After Obstetric-Related Fistula Repair
Source: Int Urogynecol J. 2025 Nov 12;37(5):1297–306. doi: 10.1007/s00192-025-06361-1 (PMC13226345; doi:10.1007/s00192-025-06361-1)
Supplement: Supplementary file 1 — Supplementary file1 (DOCX 58 KB) [file 192_2025_6361_MOESM1_ESM.docx]

**Appendix 1**

**WFF Fistula Treatment Reintegration Study**

**Pre-Surgery Questionnaire**

Interview date:

How interview was conducted:

Phone

In person

Other (Describe)

Person conducting interview:

Client first name:

Client last name:

Medical Record #:

Treatment Facility:

Expected date of surgery:

**Client Questions; questions about your life now**

1. What is your age?

2. Do you live with?

Husband

Parents

Children

Other relatives

Friends

Live alone

3. What is your current marital status?

Single

Married

Separated

Divorced

Widowed

4. What is your current occupation or business? Mark all that apply.

Housewife

Farming

Business

Laborer

Student

Unemployed

Other (Describe)

5. What financial support do you have?

Self

Husband/partner

Parents

Children

Other (Describe)

6. Which is the primary support?

Self

Husband/partner

Parents

Children

Other (Describe)

7. Do you own any land in your own name?

Yes

No

8. Do you own any income producing assets like chickens, a sewing machine or

farmland?

Yes

No

9. Have you joined any support or solidarity groups?

Yes

No

10. Have you ever used savings you may have for business or money lending?

Yes

No

11. Of your total household expenses, what proportion is met through your own

earnings?

100%

Most

About half

A small amount

None

**Questions about your fistula**

12. How long have you lived with fistula?

13. How often do you leak urine?

Never (Go to Question 15)

Once a month

A few times a month

A few times a week

Most days

All the time

14. How often do you leak urine or have stools by accident?

Never (Go to Question 16)

Rarely

Sometimes

Most of the time

15. Is the leakage?:

Mild

Moderate

Severe

16. Do you have difficulty walking?

Yes

No (Go to Question 19)

17. Why do you have difficulty walking? Mark all that apply:

Pain

Tiredness

Leg weakness

Other (Describe)

18. How severe is your problem with walking?

Mild

Moderate

Severe

19. Are you experiencing physical pain related to your fistula?

Yes

No (Go to Question 23)

20. Please describe the location of the pain and how severe it is.

21. To what extent does your pain interfere with your daily activities? A lot

A little

Not at all

22. Are you hoping to become pregnant again after your fistula surgery?

Yes

No

23. Are you using any type of birth control or family planning?

If yes, check all that apply. If no, go to Question 24

Pills

Injection

IUD device in the uterus

Diaphragm

Condom

Rhythm or timing of your cycle

None of the above (Go to Question 24)

24. Currently, how would you rate each of the following aspects of your life?

(Mark with an X; note if does not apply)


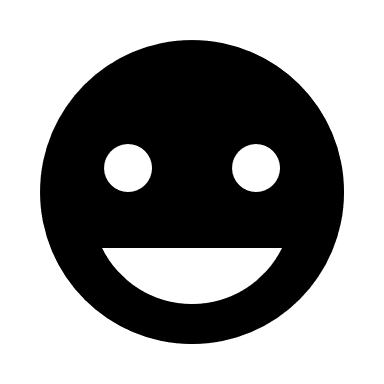

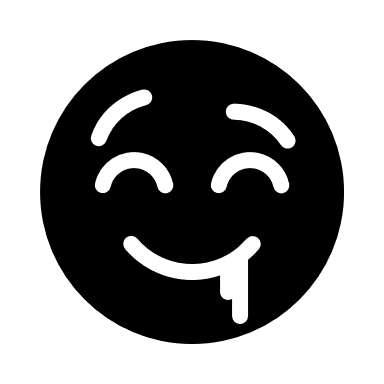

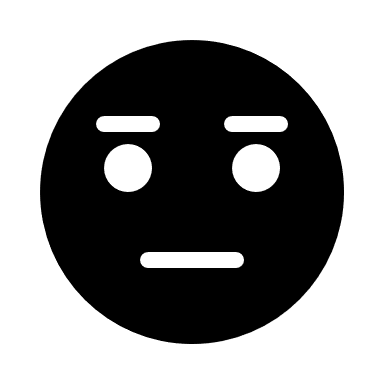

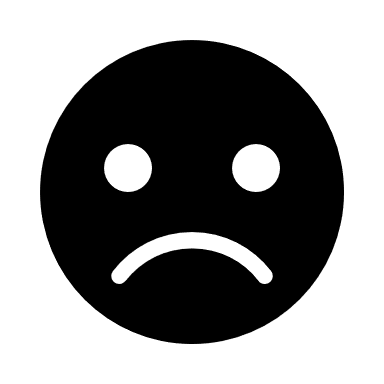


Excellent Good Fair Poor

Your marriage

Your family life

Ability to do household work like cooking

Ability to do activities outside the house like shopping

Ability to go to school or work

Ability to socialize with family and friends

Relationship with your community

25. I’m going to read a list of statements related to different ways you might feel

about yourself. Do you agree or disagree? (Mark with an X; note if does not

apply)


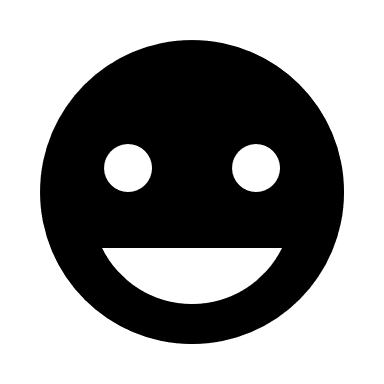

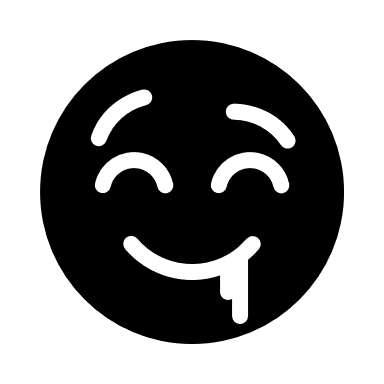

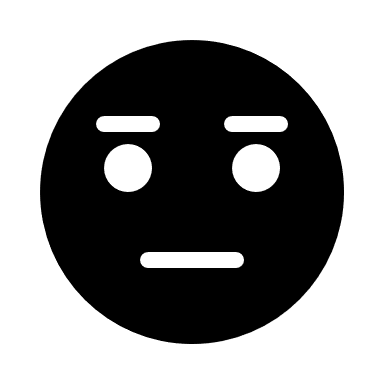

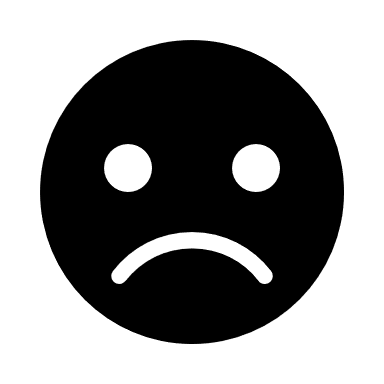


Excellent Good Fair Poor

Overall, I am satisfied with myself

At times I feel like I am no good at all

I can do things as well as most other people

I feel I do not have much to be proud of

Overall, I am inclined to feel that I am a failure

I feel that I am a person of worth at least equal with others

26. Over the past two weeks, how often have you been bothered by the following?

Not at all Several days Over half the days Nearly every day

Little interest or pleasure in doing things

Feeling down and depressed or hopeless

27. How hopeful do you feel about your future?

Very hopeful

Somewhat hopeful

Not at all hopeful

28. What do you think your biggest challenge will be when you return home after

your surgery?

**WFF Fistula treatment Reintegration Study**

**Follow up**

**Post Surgery Questionnaire**

Interview date:

How interview was conducted:

Phone

In person

Other: (Describe)

Person conducting interview:

Client first name:

Client last name:

Medical Record #:

Treatment Facility:

Date of surgery:

**Client Questions; questions about your life now**

1. Since your fistula surgery, have you received any of the following types of support? Check all that apply. Yes No

Education or learning a skill

Business training or opportunities

Financial assistance

Material needs such as food or clothing

Shelter

Family planning counseling or services

Emotional or psychological support

Joining a support or solidarity group

1. What types of support have you most benefited from? Mark up to three.

Education or learning a skill

Business training or opportunities

Financial assistance

Material needs such as food or clothing

Shelter

Family planning counseling or services

Joining a support or solidarity group

3. Do you live with?

Husband

Parents

Children

Other relatives

Friends

Live alone

4. What is your current marital status?

Single

Married

Separated

Divorced

Widowed

5. Has your economic situation changed since receiving fistula treatment?

Much better

Better

No change

Worse

Much worse

6. What is your current occupation or business? Mark all that apply.

Housewife

Farming

Business

Laborer

Student

Unemployed

Other (Describe)

7. What financial support do you have?

Self

Husband/partner

Parents

Children

Other (Describe)

8. Which is the primary support?

Self

Husband/partner

Parents

Children

Other (Describe)

9. Do you own any income producing assets like chickens, a sewing machine or

farmland?

Yes

No

10. Have you used savings you may have for business or money lending?

Yes

No

11. Of your total household expenses, what proportion is met through your own

earnings?

100%

Most

About half

A small amount

None

**Questions about your fistula and treatment**

12. Have you had a follow up visit after your fistula surgery?

Yes

No (Go to Question14)

13. Where did you get follow up?

Where I had my surgery

Another health center

14. Do you leak urine or have stools accidently now?

Yes

Never (Go to Question 18)

15. Is the leakage?

Mild

Moderate

Severe

16. Do you have difficulty walking?

Yes

No (Go to Question 23)

17. Why do you have difficulty walking? Mark all that apply.

Pain

Tiredness

Leg weakness

Other (Describe)

18. How severe is your problem with walking?

Mild

Moderate

Severe

19. Are you experiencing physical pain related to your fistula surgery?

Yes

No (Go to Question 26)

20. Is your pain

Mild

Moderate

Severe

21. To what extent does your pain interfere with your daily activities?

A lot

A little

Not at all

22. If you have any other health problems related to your fistula or fistula surgery,

please describe them.

23. Have you become pregnant since your fistula surgery?

Yes

No (Go to Question 26)

24. Have you visited a health center for antenatal care before delivery?

Yes (Where?)

No

25. Where do you expect to deliver your baby?

26. Are you hoping to become pregnant in the next few years?

Yes

No

27. Are you using any type of birth control or family planning? Check all that

apply.

Pills

Injection

IUD device in the uterus

Diaphragm

Condom

Rhythm or timing of your cycle

None of the above (Go to question 24)

28. Currently, how would you rate each of the following aspects of your life?

(Mark with an X; note if does not apply)


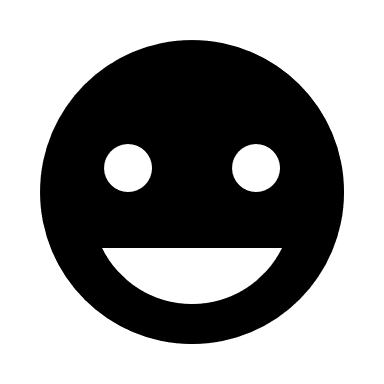

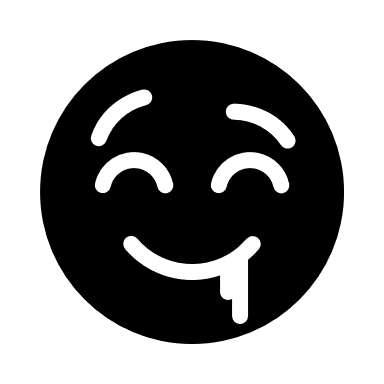

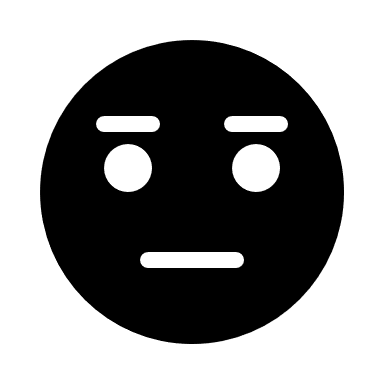

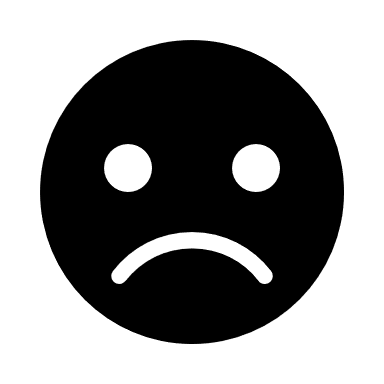


Excellent Good Fair Poor

Your marriage

Your family life

Ability to do household work like cooking

Ability to do activities outside the house like shopping

Ability to go to school or work

Ability to socialize with family and friends

Relationship with your community

29. I’m going to read a list of statements related to different ways you might feel

about yourself. Do you agree or disagree?

Yes No

Overall, I am satisfied with myself

At times I feel like I am no good at all

I can do things as well as most other people

I feel I do not have much to be proud of

Overall, I am inclined to feel that I am a failure

I feel that I am a person of worth at least equal with others

30. Over the past two weeks, how often have you been bothered by the following?

Not at all Several days Over half the days Nearly every day

Little interest or pleasure in doing things

Feeling down and depressed or hopeless

31. How hopeful do you feel about your future?

Very hopeful

Somewhat hopeful

Not at all hopeful

32. What was your biggest challenge when you returned home after your surgery?

33. What to what types of support would be helpful to you now?
